# A narrative review on the effects of a ketogenic diet on patients with Alzheimer's disease

**DOI:** 10.3934/publichealth.2022014

**Published:** 2021-12-22

**Authors:** Ethan Ali Tabaie, Akshay Jakkidi Reddy, Hetal Brahmbhatt

**Affiliations:** 1 Department of Neurobiology, Physiology & Behavior, University of California, Davis, Davis, USA; 2 Department of Ophthalmology, California Northstate University College of Medicine, Elk Grove, USA; 3 Psychiatry, Mercy General Hospital, Sacramento, USA

**Keywords:** ketogenic diet, Alzheimer's disease, low-carbohydrate, cognition

## Abstract

Alzheimer's disease (AD) has been very difficult to prevent and cure using the medicine available today. However, there has been some hope with using a ketogenic diet (KD) to reduce the cognitive and quality of life decline experienced by patients with AD. In this review, the authors discuss the research done on the effect of a KD on AD to provide some potential avenues for future research and to determine a KD that can be best adopted by patients. The authors also go over the effects of KD's and low-carbohydrate diets (LCDs) on the cognitive function of healthy patients and on patients without AD to determine the similar and dissimilar effects of the diets. The authors found that the KD was able to improve the cognitive abilities and quality of life of patients ranging from mild to severe AD. Several types of memory were improved as a result of the diets. Further research needs to be conducted to determine the cause behind these improvements. However, the several studies that were done were mostly in agreement that once ketosis was reached, cognitive improvements were observed in patients ranging from mild to severe AD or mild to moderate cognitive impairment. Through the use of a KD, potential mechanisms can be found to reduce the cognitive decline of patients with AD, and potentially even prevent the damaging effects of cognitive decline from AD altogether.

## 1. Introduction

Alzheimer's disease (AD) is currently the sixth-leading cause of death in the U.S. [1]. Around 6.2 million Americans are living with the disease in 2021, and about eleven percent of the population over 65 years of age have the disease [1]. Advancements have been made on reducing and eventually preventing the effects of AD, and the one effect focused on in this paper is cognitive decline. Ketogenic diets are currently being studied as a method to help reduce or prevent the onset of cognitive decline in Alzheimer's patients [2].

The ketogenic diet's goal is to reduce one's reliance on using glucose as fuel for the brain and instead use ketones [2]. Since AD could potentially reduce the number and overall function of glucose transporters in the brain [3], ketones could provide an alternate avenue to supply the brain with energy in patients with AD. The studies on the several diets that induce ketosis have some discrepancies. The purpose of this paper is to determine the best ketogenic diet that has been tested currently for preventing the harmful effects of AD. This paper looks at the cognitive improvements associated with the different diets. For example, whether medium-chain triglycerides are used and the effects of using a ketogenic diet and not another diet that does not achieve ketosis.

While prevention of Alzheimer's disease may be very far in the future, our effort to reduce its prevalence in the population will undoubtedly benefit the world and future families that could suffer from the disease. This review is set apart from others because it looks at the cognitive effects of a KD and LCD on people without AD as a method of comparison.

## 2. Methods

Randomized controlled trials were searched using PubMed with the search terms “(((Alzheimer's) AND (ketogenic diet) AND (cognitive function)) OR ((ketogenic diet) AND (cognitive function)) OR ((low-carbohydrate diet) AND (cognitive function)))”. Only articles in English were included in the analysis, and papers not focusing on the cognitive changes within human patients while on a low-carbohydrate diet were not included. The 1995 paper by Wing et al. was not included as only an abstract could be found. Only literature published online within PubMed was included in the review. Using the search terms and criteria mentioned, only 21 of the 25 papers found were used in the literature review.

## 3. Discussion and results

### 3.1. The effect of ketosis on Alzheimer's disease progression

Ketosis, which is defined by having a blood ketone concentration of 0.5–0.6 mmol/L [4], has been achieved in the two studies where cognitive improvements were seen after consumption of a KD according to Table 1 [5],[6]. Both diets used medium-chain triglycerides (MCTs) as a ketogenic factor [5],[6]. Both studies looked at Mild AD patients, but the Mediterranean Diet with Coconut Oil was also given to patients with moderate and severe AD while still showing cognitive improvements especially in the severe AD patients [6].

Though these two studies show a connection between ketosis and cognitive improvements in Alzheimer's patients, two different studies showed no cognitive improvements in patients suffering from Mild and Mild -Moderate AD [4],[7]. In the Henderson et al. study, a ketone body increase was not seen in the participants of the study following the caprylic triglyceride formula meant to induce ketosis according to Table 1 [7]. The Phillips et al. paper did achieve ketosis in its patients by using a more classical ketogenic LCD; however, it did not show an increase in cognitive function of patients with mild AD [4]. Therefore, this paper goes against the belief that ketosis induces cognitive improvements in Alzheimer's patients. Since only one paper shows no connection between ketosis and improving cognitive function in AD patients, there must be more studies done to determine the true effect of the diet. Another thing to keep in mind, the two papers that improved cognitive function used a modified Atkins diet with MCTs and a Mediterranean Diet with Coconut Oil, which has MCTs [5],[6]. Therefore, MCTs may be the factor in improving cognition in AD patients, so that can be something tested for in the future. However, the studying using an LCD went into the COVID-19 lockdown, and the authors suspect some cognitive burden caused by the seclusion could have affected the upward trend in cognitive improvements [4]. Once again more studies need to be done to determine whether the MCTs in the ketogenic diet caused the cognitive improvements in AD patients or whether it was the ketosis itself.

The study where a 40-gram dose of caprylidene was used showed an increase in ketone body levels in patients according to Table 1, but the experimental procedure did not include a cognitive test for memory similar to the other studies described [8]. However, blood flow to several different areas of the brain was measured and significantly increased following the 45-day diet [8], which could indicate that a future study might improve the cognitive function of patients if increasing the blood flow to the brain increases its cognitive abilities. This was also the successful use of a caprylidene diet in inducing ketosis in participants while the 50% caprylic triglyceride AC-1204 Formula did not according to Table 1.

There is an issue with assuming that all AD patients will react equally with the ketogenic diets as patients with *APOE e4* alleles were excluded or when included showed little statistical significance between cognitive improvements and ketone body levels. The study using a 40-gram dose of Caprylidene even showed no changes in cerebral blood flow experienced by the subjects with AD and an *APOE e4* allele [8]. As a result, the potential positive effects of a KD on AD patients might be limited to those without the *APOE e4* allele.

**Table 1.** Methodological analysis of ketogenic and low-carbohydrate diets within the literature review.

Author (year)	Experimental Diet Used (percentage of total calories)	Presence of MCTs in Diet	Dropout Rate of Low-carb Diet	Patient Population Tested	Presence of Ketone Body Increase	Cognitive Improvements Observed
Brandt (2019) [5]	Modified Atkins with MCTs	Yes	20	Mild AD	Yes	Yes
Brinkworth (2009) [15]	LCD (61% Fat, 35% Protein, 4% Carb)	No	58	Overweight or Obese Adults	N/A	No
de la Rubia Orti (2018) [6]	Mediterranean Diet with Coconut Oil (30% Fat, 15% Protein, 55% Carbs)	Yes	N/A	Mild-Moderate AD and Sever AD	Yes	Yes
El-Rashidy (2017) [9]	Modified Atkins with MCTs (60% Fat, 30% Protein, 10% Carb)	No	33.3	Children With Autism Spectrum Disorder	Yes	Yes
Emilien (2017) [11]	High Protein LCD (30% Fat, 40% Protein, 30% Carb)	No	0	Healthy Adults	N/A	No
Fischer (2004) [12]	LCD (100% Fat) and LCD (100% Protein)	No	N/A	Healthy Adult Men	N/A	No
Fortier (2021) [20]	KD with MCTs	Yes	38	Adults and Mild Cognitive Impairment	Yes	Yes
Hlayburton (2007) [17]	LCD (61% Fat, 35% Protein, 4% Carb)	No	7.69	Overweight or Obese Adults	Yes	Yes
Henderson (2020) [7]	50% Caprylic Triglyceride AC-1204 Formula	Yes	24.3	Mild-Moderate AD	No	No
Holloway (2011) [10]	LCD (73.6% Fat, 24.6% Protein, 3.6% Carb)	No	N/A	Healthy Adult Men	N/A	No
Iacovides (2019) [9]	LCD (60% Fat, 25% Protein, 15% Carb)	No	8.3	Healthy Adults	Yes	No
IJff (2016) [18]	LCD (61% Fat, 20% Protein, 20% Carbs) and KD with MCTs (Fat is 30% MCTs)	Yes	0	Children and Adolescents with Refractory epilepsy	N/A	Yes
Kakoschke (2021) [16]	LCD (58% Fat, 28% Protein, 14% Carb)	No	3.45	Obese Adults with T2DM	N/A	No
Karl (2015) [13]	LCD (30% Fat, 42% Protein, 28% Carb)	No	0	Healthy Adults	N/A	No
Lee (2021) [22]	KD with MCTs (37.3% Fat, 15.7% Protein, 47.0% Carb)	Yes	0	Adults with Primary Progressive Multiple Sclerosis	Yes	No
Markus (1999) [23]	Protein Rich Carbohydrate Poor Diet (32% Fat, 27% Protein, 41% Carb)	No	0	High and Low Stress Prone Students	N/A	No
Morrison (2020) [21]	LCD (68.2% Fat, 24.4% Protein, 7.3% Carb)	No	0	Adults with HIV and Mild-to-Moderate Cognitive Impairment	Yes	Yes
Phillips (2021) [4]	LCD (58% Fat, 29% Protein, 7% Net Carb)	No	19	Mild AD	Yes	No
Torosyan (2021) [4]	40g Caprylidence	Yes	29	Mild-Moderate AD	Yes	N/A
Yomogida (2021) [14]	KD with MCTs (87.16% Fat (48.31% MCTs, 38.83% LCTs), 8.09% Protein, 4.75% Carb)	Yes	N/A	Healthy Elderly Individuals	Yes	Yes

### 3.2. The effect of ketogenic and low carb diets on patients other than AD patients

To study the effects of low-carb and ketogenic diets on KD patients, we also looked at its effects on other patient populations. Healthy adults were given low-carb diets in five studies according to Table 1. All five studies showed no cognitive improvements after consuming the low-carb diets [9]–[13]. The Iacovides et al. study also induced ketosis in the participants by having a blood β-hydroxybutyrate value above 0.4 mmol/L [9]. Therefore, the presence of ketone bodies at ketosis values still did not improve the cognition of healthy participants. As a result, healthy adults may not be affected cognitively by ketosis or by LCDs. However, since no diet tested on healthy adult participants contained MCTs, the connection between the consumption of MCTs and improved cognition was not challenged.

Healthy elderly participants consuming an LCD with MCTs did experience an increase in ketone bodies to ketosis levels described in the study as plasma ketone levels of 0.3–0.5 mM [14]. Their cognitive function was also improved as a result of the ketone bodies [14] meaning that elderly individuals could potentially benefit from consuming MCTs and inducing ketosis.

Since patients who have Alzheimer's tend to not have effective glucose transporters within the brain [3], a diet high in fat or ketones, an alternative energy source for the brain, could be more effective at reducing the brain's oxidative burden from lacking energy. This could potentially be tested by observing the cognitive changes of diets that induce ketosis in patients with diabetes.

We looked at three different papers that administered LCDs to overweight or obese adults according to Table 1 and found two of the three showing no cognitive improvements [15],[16]. The two that showed no changes were both tested on only overweight or obese adults while the third study determined an increase in ketone bodies and showed cognitive improvements in obese adults with type two diabetes mellitus (T2DM) [17]. This furthers the hypothesis that dysfunctional glucose transporters could cause cognitive decline in AD patients since the presence of a ketone body increase in patients with T2DM also show improvements in cognitive functions as well.

According to Table 1, four other studies also showed cognitive improvements after consuming an LCD or a KD with MCTs [18]–[21]. The study testing a four-month KD on children and adolescents with refractory epilepsy showed improved cognitive activation and lower levels of anxious behaviors [18]. Patients with mild cognitive impairment associated with and not associated with HIV also showed improvements in cognitive functions after consumption of a KD [20],[21]. Finally, children with autism spectrum disorder also experienced improved cognitive results [19]. Therefore, the mechanism by which cognitive function is improved through a KD could be found in healthy elderly patients but also patients with these variety of cognitive disorders. These mechanisms may not be found in adults with primary progressive multiple sclerosis or stress-prone students as neither showed any cognitive effects due to the low-carb or KD diets [22],[23].

There are many other cognitive and metabolic conditions that a ketogenic diet could be tested with to determine the chemical pathways being affected. Therefore, further testing still needs to be done to determine the cause for the discrepancies between the many different diets in the study.

### 3.3. Problems with dropout

It is essential for diets used in the future that induce KD and result in an improvement in cognition among AD patients to be as easy to implement within a real-world application. There were several differences in retention that were seen in the studies that introduced KDs that improved the cognition and quality of life for patients with AD. For instance, the modified Atkins diet in the Brandt et al. study showed a high rate of dropout compared to the other diets [5] while the Mediterranean diet with coconut oil had no mention of dropout [6]. In the Brandt et al. study, the modified Atkins diet (MAD) used showed similar dropout rates to other studies with the ketogenic Atkins diet [5] showing that the diet, in general, has difficult retention amongst patients with AD or cognitive impairments. The other MAD used in the study also had an above-average dropout rate of 33.3% mostly due to poor compliance with the diet [19].

Three of the studies using ketogenic diets with MCTs where dropout was recorded according to Table 1 stated that most of the dropouts were due to the gastrointestinal issues caused by ingesting MCTs, which is well known [5],[7],[20]. Since many of these studies did not take a detailed look into the desire of patients to use the diets, there needs to be much more research on the practicality of using a KD for AD patients. However, using high amounts of MCTs in a KD might cause gastrointestinal issues, so that should be a consideration when searching for a useful and practical KD diet.

The authors recommend using a longitudinal study of patients with and without increased risk of developing AD and giving them an easily attainable KD to not only witness any biological changes but also to determine whether there was consistent avoidance of AD much higher than the expected amount from this population. Therefore, the study that needs to be done must be a large study including a large population of individuals as well as a study using a known diet that induces ketogenesis with high adherence.

## 4. Conclusions

Through this study, a KD has the potential to improve the cognitive function of patients with AD and improve their quality of life if ketosis was achieved. Throughout the studies, cognitive improvements were only seen until the ketone levels of the patients were increased, therefore, indicating that ketosis is the probable factor that improves cognitive function in the patients. However, more studies must be done to determine whether or not a ketogenic diet is actually the culprit for improving cognition in AD patients. The authors propose a longitudinal study that will have two groups of participants: one consuming a KD and one consuming a control normal diet. This study will then focus on collecting data regarding the health and cognitive faculties of the individuals. To further improve the study, one could also break up these groups into two categories: at risk for Alzheimer's disease and not at risk for Alzheimer's disease. The authors believe taking an in-depth biological analysis of these individuals will be useful in determining the true cause of the improvements in cognition and whether or not they are due to the ketogenic diet. Although further studies must be done, the current data compels us to believe that ketosis at least has some effect on improving the quality of life and cognition of patients with AD.
